# Supplementary material for: Effects of Dietary Ferulic Acid on Intestinal Health and Ileal Microbiota of Tianfu Broilers Challenged with Lipopolysaccharide
Source: Molecules. 2023 Feb 10;28(4):1720. doi: 10.3390/molecules28041720 (PMC9967589; doi:10.3390/molecules28041720)
Supplement: Supplementary file 1 [file molecules-28-01720-s001.zip › molecules-2169277-supplementary.pdf]

Effects of Dietary Ferulic Acid on Intestinal Health and Ileal Microbiota of Tianfu Broilers Challenged with Lipopolysaccharide

Ziting Tang [1,2,†], Gang Shu [1,†], Hong Du [1], Yilei Zheng [3], Hualin Fu [1], Wei Zhang [1], Cheng Lv [1], Funeng Xu [1], Haohuan Li [1], Ping Ouyang [1], Juchun Lin [1], Li-Jen Chang [4], Felix Kwame Amevor [1,5] and Xiaoling Zhao [1,5,*]

Supplementary Materials

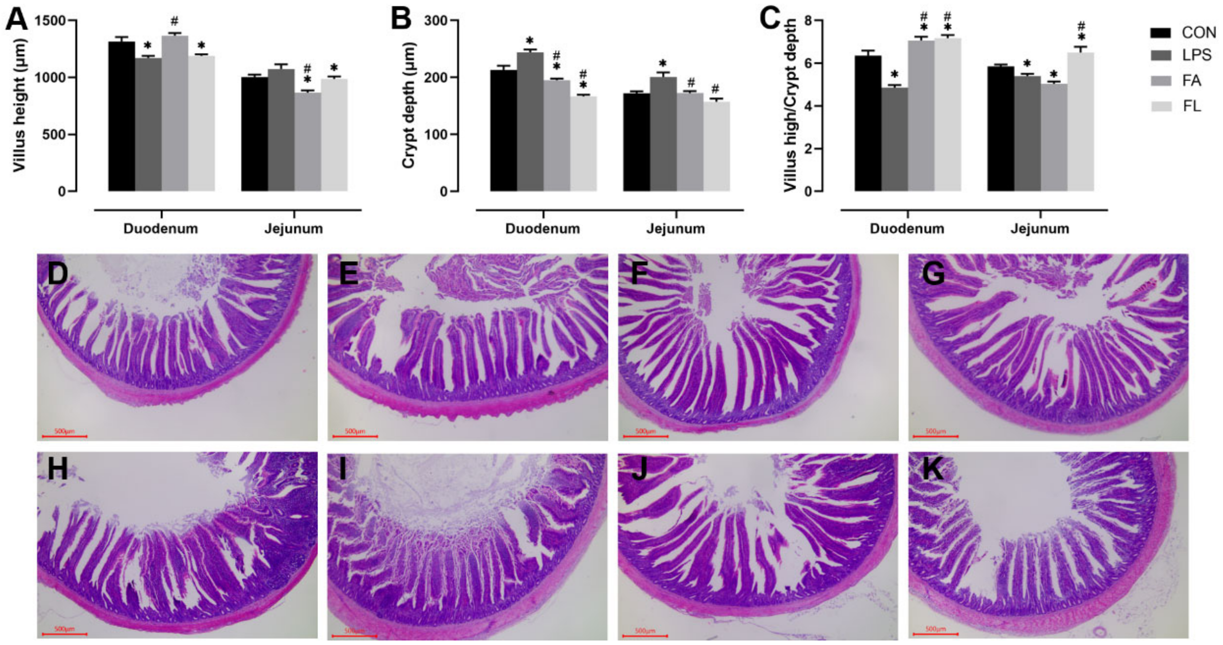

Figure S1. The data and histology of duodenum and jejunum. (**A**) villus height (μm). (**B**) Crypt depth (μm). (**C**) Villus height / Crypt depth ratio. Values were shown in means±SEM. *$p < 0.05$ compared with the CON group. # $p < 0.05$ compared with the LPS group. (**D-K**) The representative histology of the duodenum and jejunum showing villus height and crypt depth with HE staining (scale bar = 500 μm). (**D**) duodenum of CON group, (**E**) duodenum of LPS group, (**F**) duodenum of FA group, (**G**) duodenum of FL group. (**H**) jejunum of CON group, (**I**) jejunum of LPS group, (**J**) jejunum of FA group, (**K**) jejunum of FL group.
